# 1725. National Surveillance of Gram-negative Bacteria in Qatar, the Study for Monitoring of Antimicrobial Resistance Trends (SMART): from 2017 to 2019

**DOI:** 10.1093/ofid/ofac492.1355

**Published:** 2022-12-15

**Authors:** Mazen A Sid Ahmed, Thoraya M Saleh, Mohamed Albirair, Khalid M Dousa, Lolita C Arisgado, Muna A Al Maslamani, Abdul Latif Al Khal, Emad B Ibrahim, Hamad Abdel Hadi

**Affiliations:** Philadelphia's Department of Public Health, Philadelphia, Pennsylvania; Hamad Medical Corporation, Doha, Ad Dawhah, Qatar; University of Washington, Seattle, Washington; Cleveland Medical Center, Cleveland, Ohio; Hamad Medical Corporation, Doha, Ad Dawhah, Qatar; HMC, doha, Ad Dawhah, Qatar; Hamad Medical Corporation, Doha, Ad Dawhah, Qatar; Hamad Medical Corporation, Doha, Ad Dawhah, Qatar; Hamad Medical Corporation, Doha, Ad Dawhah, Qatar

## Abstract

**Background:**

Monitoring Antimicrobial Resistance Trends (SMART) is a global study for the surveillance of antimicrobial resistance (AMR) in Gram-negative bacteria (GNB) from different regions around the World including Gulf countries. To evaluate prevalence and trends in AMR in GNB from clinical specimens including microbiological and genomic characteristics as well as examine Antimicrobial Susceptibility Tests (AST) for existing and novel antimicrobials.

**Methods:**

A prospective study was conducted on clinical specimens from Hamad Medical Corporation, Qatar, between 2017 and 2019 according to the SMART protocol that covers community as well as hospital-associated infections reported to secondary care. Consecutive GNB were included from different sites including lower respiratory and urinary tracts, intrabdominal, and bloodstream infections.

**Results:**

Over the three years study period, 895 isolates were studied from the specified sites comprising 38 GNB with four key pathogens: *Escherichia coli*, *Klebsiella pneumoniae*, *Pseudomonas aeruginosa,* and *Stenotrophomonas maltophilia.* Meropenem resistance was 3.6% for *E. coli* and 9.3% for *K. pneumoniae* while imipenem/relebactam resistance was 3% compared to 8% respectively. The overall ceftolozane/tazobactam resistance for *E. coli* was 9% (25/281) compared to 13% (34/257) for *K. pneumoniae* while resistance for ceftazidime/avibactam was 2% (3/137) and 4% (5/117) respectively. Genomic characteristics of 70 Enterobacterales including 48 carbapenem-resistant, revealed the prevalence of β-lactamase from all classes dominated by *bla*_CXM-15_ while carbapenem resistance revealed a paucity of *bla*_KPC_ and supremacy of *bla*_OXA-48_ like and *bla*_NDM_ resistance genes (Table 1).

**Table 1.**
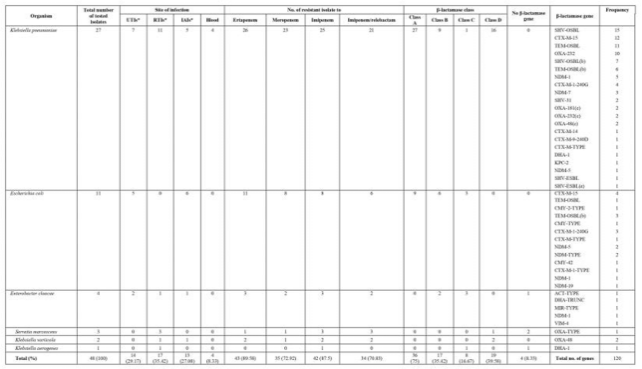
The frequency of different β-lactamase genes among 48 Carbapenem Resistant Enterobacterales (ERSs) isolates collect from Qatar between 2017 - 2019.

**Conclusion:**

Surveillance of GNB from Qatar showed the prevalence of key pathogens similar to the rest of the world but demonstrated significant resistance to existing and novel antimicrobials, particularly for *K. pneumoniae* with different underlying resistance mechanisms.

**Disclosures:**

**All Authors**: No reported disclosures.

**Table 1.** The frequency of different β-lactamase genes among 48 Carbapenem Resistant Enterobacterales (ERSs) isolates collect from Qatar between 2017 - 2019.

*Intra-abdominal infections (IAIs), respiratory tract infections (RTIs), and urinary tract infections (UTIs)

